# PROTOCOL: Effectiveness of nutrition counselling for pregnant women in low‐ and middle‐income countries to improve maternal, infant and child behavioural, nutritional and health outcomes: A systematic review

**DOI:** 10.1002/cl2.1202

**Published:** 2021-11-09

**Authors:** Omar Dewidar, Ammar Saad, Aqeel Baqar, Jessica C. John, Alison Riddle, Erika Ota, Jacqueline K. Kung'u, Mandana Arabi, Manoj K. Raut, Seth S. Klobodu, Sarah Rowe, Jennifer Hatchard, Jennifer Busch‐Hallen, Chowdhury S. B. Jalal, Sara Wuehler, Vivian Welch

**Affiliations:** ^1^ School of Epidemiology and Public Health University of Ottawa Ottawa Ontario Canada; ^2^ University of Ottawa/Bruyère Research Institute Ottawa Ontario Canada; ^3^ Faculty of Medicine, School of Epidemiology and Public Health University of Ottawa Stittsville Ontario Canada; ^4^ Eat, Drink and Be Healthy Tunapuna Trinidad and Tobago; ^5^ Faculty of Medicine, School of Epidemiology and Public Health University of Ottawa Marmora Ontario Canada; ^6^ Global Health Nursing, Graduate School of Nursing Science St. Luke's International University Chuo‐ku Japan; ^7^ Nutrition International‐Africa Regional Office Nairobi Nairobi Kenya; ^8^ Nutrition International New York New York USA; ^9^ Nutrition International New Delhi India; ^10^ Department of Nutrition and Food Science California State University, Chico Chico California USA; ^11^ Nutrition International Ottawa Ontario Canada; ^12^ Global Technical Services Nutrition International Ottawa Ontario Canada; ^13^ Methods Centre, Bruyère Research Institute Ottawa Ontario Canada

## Abstract

The objective of this systematic review is to identify, appraise and synthesise the best available evidence on the effectiveness of nutritional counselling and education interventions on maternal, infant and child health outcomes, and assess the differences in effects across participants' PROGRESS+ characteristics. To achieve these objectives, we will aim to answer the following research questions: What is the effectiveness of nutrition counselling interventions for pregnant women in low‐ or middle‐income countries on maternal, infant and child health outcomes? What are the impacts of nutrition counselling interventions on maternal, infant and child health outcomes across participants' PROGRESS+ characteristics?

## 1 BACKGROUND

### 1.1 Description of the condition

A healthy diet is necessary for a positive pregnancy experience. Providing pregnant women with proper antenatal care throughout pregnancy is a necessary component of a successful delivery. Evidence shows that foetal growth and maternal physical and mental health equilibrium require increased nutritional demands (Mousa et al., 2019). However, nutritional deficiencies are highly prevalent among pregnant women, specifically in low‐ and middle‐income countries, leading to preventable adverse maternal and birth outcomes (Darnton‐Hill & Mkparu, 2015; Viswanathan et al., 2008). Observational studies have indicated that gestational weight gain and energy intake are strongly associated with better birth outcomes, especially in undernourished women (Institute of Medicine, 1990; Kramer et al., 2008; Kramer, 1987; Rush, 2001). Substantial evidence exists to support that nutrition counselling, in the presence or absence of other communication channels and tools carries the potential to improve nutrition practices and health outcomes, in part, through health education and promotion (Bhutta et al., 2008, 2013; Graziose et al., 2018; Mbuagbaw et al., 2015; Tekelab et al., 2019; World Health Organization [WHO], 2016). In response to these and similar concerns, the WHO has recommended that “Counselling on healthy eating and physical activity” be integral to women's antenatal care (WHO, 2016). Despite the potential power of nutrition counselling, limited time, infrastructure, staff capacity and motivation often hinder or prevent the delivery of quality antenatal care in low‐resource settings—and often, nutrition counselling does not happen or does not happen well (Girard & Olude, 2012). This is important because many evidence‐based nutrition actions achieve the desired outcomes when women and mothers use a recommended nutrition practice at home. Furthermore, if a supportive enabling environment is in place (e.g., maternal micronutrient supplements are available at distribution points in adequate quantities and quality, supportive policies, adequately compensated and distributed health staff, etc.) quality counselling with beneficiaries can improve provider job satisfaction, retention, and ability to provide nutrition services to a higher quality standard (Girard & Olude, 2012; Sunguya, Poudel, Mlunde, Shakya, et al., 2013; Sunguya, Poudel, Mlunde, Urassa, et al., 2013). A better understanding of the current coverage of interpersonal nutrition counselling during antenatal care in LMICs can help better target resources and advocacy for accelerated progress toward the sustainable development goals (SDGs).

Pregnant women in low‐ or middle‐income countries are at an increased risk of facing unjust or unfair health disparities, especially during the critical time of their pregnancy. The systematic social disadvantage associated with their status jeopardises the quality of the antenatal care they receive. As well, their health inequities are further magnified by the disadvantages they experience due to their Place of residence, Race and culture, Occupation, Gender and sex, Religion, Education, Socioeconomic status, Social capital, plus: personal characteristics (i.e., age, disabilities), relationship features (i.e., exclusion from school, parent drug use), and time‐dependent relationships (i.e., leaving the hospital or other times when an individual might be temporarily disadvantaged). Such characteristics are better known using the acronym PROGRESS+ and are useful to identify differences in health outcomes among socially disadvantaged populations (O'Neill et al., 2014).

### 1.2 Description of the intervention

Nutrition counselling interventions have varied considerably over the years (Vasiloglou et al., 2019), therefore we defined the intervention as follows based on the World Food Programme (World Food Programme, 2014): “Counselling provided to individuals or in group sessions, that includes two‐way interactive education linked to promoting specific behaviours.” There is growing interest in identifying the effectiveness of nutrition counselling interventions on postpartum women's health and behavioural outcomes as well as neonates' health outcomes. The aim of this review is to investigate the effectiveness of the nutrition counselling interventions to improve maternal and child nutrition and health in low‐ and middle‐income countries.

### 1.3 How the intervention might work

Women's empowerment is positively associated with improved health and nutrition outcomes for women and children (Carlson et al., 2015; Cunningham et al., 2015; Pratley, 2016). Nutrition counselling programmes that incorporate an empowerment approach, as with a minimum of two‐way interactive education, may improve intervention uptake and effectiveness by increasing women's urgency to act on the information provided through nutrition counselling sessions. In turn, the programme can create a supportive opportunity structure (Alsop Ruth Heinsohn Nina, 2005) where women can access the necessary material, financial and social supports needed to affect positive change. We have adapted the model used by Riddle *et al*. to reflect the causal pathways through which an empowerment approach may contribute to improved health and nutrition outcomes for pregnant women and their infants. We adopt the definition of empowerment developed by Kabeer (1999): “the expansion in people's ability to make strategic life choices in a context where this ability was previously denied to them”.

As part of this review we will look for evidence of nutrition counselling interventions that apply a complete empowerment model. We define a complete empowerment model as including two components as outlined in Riddle et al.: (1) Nutrition counselling to foster agency and (2) activities to create a supportive opportunity structure. Nutrition counselling interventions that foster agency will provide participants with a “space for self‐reflection and identification of important life areas” in relation to their nutritional status (Shankar et al., 2019).

In other words, the counselling intervention provides opportunities for participants to identify and assess barriers to improving their nutrition and identifying goals and opportunities to improve their nutritional status. In turn, this is expected to increase their motivation for behaviour change and self‐efficacy. Activities to create a supportive opportunity structure will provide participants with the necessary material, social or financial resources needed to act on the knowledge and skills they acquire through nutrition counselling (Alsop Ruth Heinsohn Nina, 2005; Kabeer, 1999). This includes altering the constraining political, economic, sociocultural, intrafamilial or legal structures (both formal and informal) that can limit behaviour change (Alsop Ruth Heinsohn Nina, 2005; Mahotra Anju & Boender, 2002). Examples of opportunity structure‐related activities are engaging participants' families in nutrition counselling to increase their awareness and support for proper nutrition during pregnancy or providing financial support to participants to increase their access to food or health services.^1^

Nutritional health education interventions provide pregnant women with nutritional counselling and help reduce the risk of developing nutritional‐specific complications, especially during labour and delivery (Girard & Olude, 2012). The common characteristic of such interventions lies in the provision of direct or indirect communication focusing on influencing their knowledge about the importance of a sufficient and balanced diet during pregnancy, and improving their behaviours and attitudes towards the use of necessary nutritional supplements such as fortified foods and vitamins when appropriate (Arrish et al., 2014). Nutrition counselling for pregnant women also seeks to influence their behaviour with regard to accessing quality antenatal care services, promoting delivery at the health facility, and accessing quality postnatal care services (Alam et al., 2005; Girard & Olude, 2012; Perumal et al., 2013).

**Figure 1:**
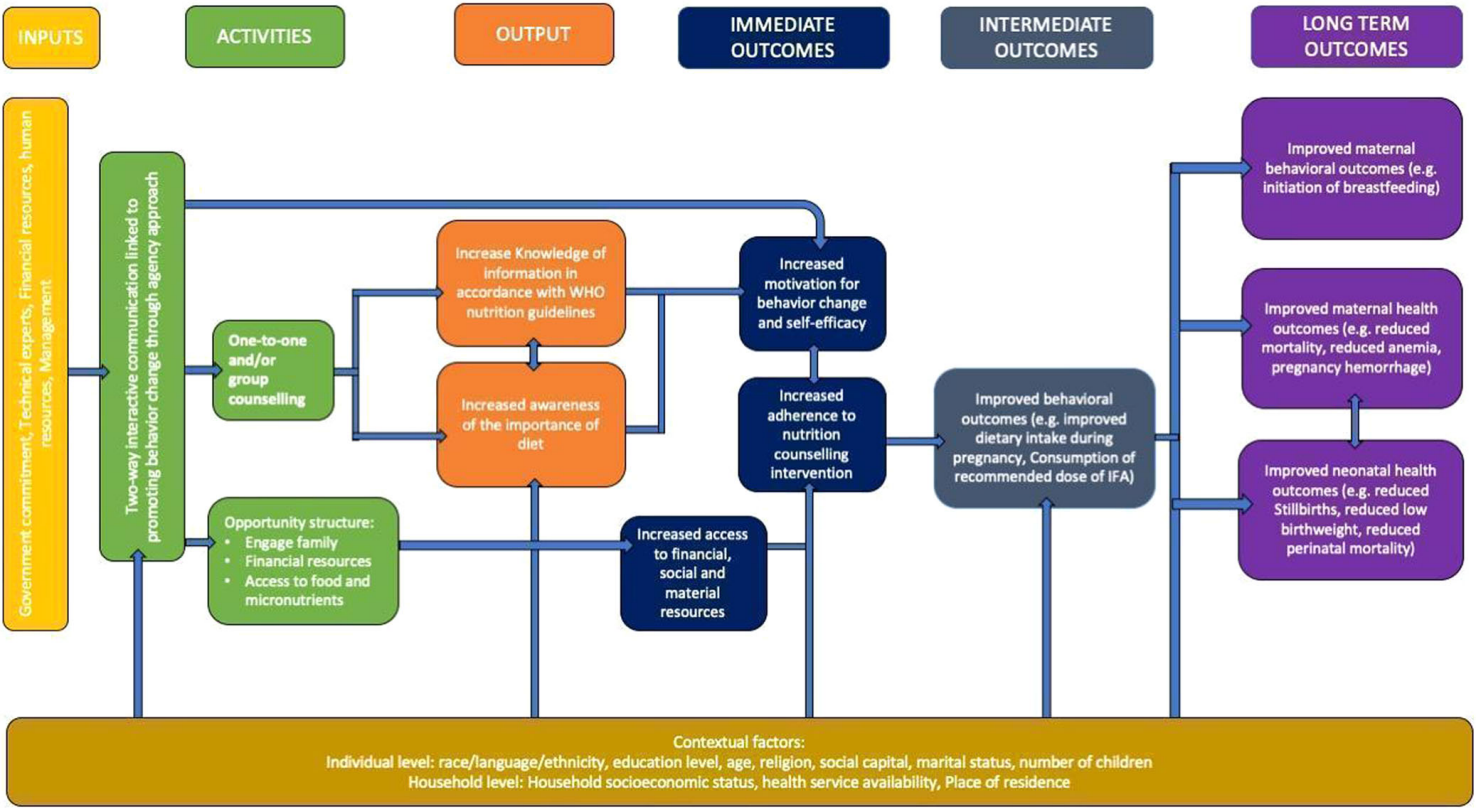
Logic model for nutrition counselling intervention to improve maternal and neonatal behavioural and health outcomes

We hypothesise that nutrition counselling interventions for 2–4 months, conducted through weekly home visits and group meetings while covering the importance of increasing food quantities and improving food quality, would increase the women's knowledge of nutrition‐related information according to international practice guidelines. Trained providers must provide this information, particularly when empowerment‐based, that is, counselling accompanied by nutritional supplements. In the process, women will also gain awareness on the importance of prenatal and postpartum nutrition. Peer support in the form of family and friends in the counselling sessions' group sessions may improve adherence to the sessions. Thus, we hypothesise that nutrition counselling would lead to increased motivation and resource support for behaviour change, which would translate into increased adherence to the nutrition counselling intervention. In the intermediate term, these immediate outcomes would improve behavioural outcomes such as dietary intake and diversity during pregnancy. In the long term, nutrition counselling may lead to improved maternal and neonatal health outcomes. These may reduce mortality, pregnancy complications, anaemia, stillbirths and perinatal mortality (see Figure 1 for logic model). The provided descriptions are not a comprehensive list of the possible methods for conducting nutrition counselling. We aim to collect the characteristics of the counselling programmes to determine the most effective structure for nutrition counselling.

Currently, we acknowledge there are also several factors at an individual and household level that may mediate the effectiveness of the intervention, as well as the suggested outcomes along the causal pathway (Pouchieu et al., 2013). These include a household's socioeconomic status or an individual's level of education. We will apply the PROGRESS+ framework (O'Neill et al., 2014) to identify these socially stratifying factors and where possible, conduct subgroup analyses to look at the differences in effects by group.

### 1.4 Why it is important to do this review

There is limited evidence on the effectiveness of nutritional education and counselling interventions among pregnant women; a Cochrane systematic review found that such interventions tended to lower the risk of preterm delivery, but had no effect on neonatal complications or stillbirth (Ota et al., 2015). A similar meta‐analysis of controlled experimental trials found a significant association between receiving nutritional counselling and improved gestational and birth weight, as well as reduced risk of anaemia among pregnant mothers (Girard & Olude, 2012). Even though both reviews have shown that nutritional education and counselling carry the potential to positively influence the health status of both the mother and the infant, several methodological limitations in the breadth of their searches and selection of study designs prevented a comprehensive synthesis of knowledge around the effectiveness of such interventions. To the best of our knowledge, our systematic review will provide the most robust and equity‐focused analysis on the effectiveness of nutritional education and counselling among pregnant women in low‐ or middle‐income countries for these health and nutrition outcomes analysed by the previous review as well as an analysis of behavioural outcomes.

While nutrition counselling and education has been shown to be effective among pregnant women in high income countries (Goodarzi‐Khoigani et al., 2018), the impact of such interventions on maternal and foetal health outcomes in low‐ or middle‐ income countries have not been well examined or documented. Moreover, little evidence is available on the implications such interventions might carry on the health equity of pregnant women in such socially and economically disadvantaged settingsrepresented by PROGRESS+ characteristics. The added value of our systematic review is to not only provide a comprehensive analysis of the effectiveness of nutritional counselling and education interventions, but also to use an equity lens in interpreting the differences in the magnitude of their effectiveness across participant characteristics. This review comes in response to the need to implement interventions capable of decreasing nutritional‐related morbidity and mortality rates and address the global health issue that is part of the SDGs set by the United Nations (Girard & Olude, 2012). Consequently, the World Health Organization has recently recommended the use of nutritional counselling in improving healthy eating habits of pregnant women (WHO, 2019). The results of this review will help inform patients and policy makers about effective and equitable measures to improve the health status of pregnant women and their infants in countries where malnutrition is highly prevalent.

## 2 OBJECTIVES

The objective of this systematic review is to identify, appraise and synthesise the best available evidence on the effectiveness of nutritional counselling and education interventions on maternal, infant and child health outcomes, and assess the differences in effects across participants' PROGRESS+ characteristics.

To achieve these objectives, we will aim to answer the following research questions:

- What is the effectiveness of nutrition counselling interventions for pregnant women in low‐ or middle‐income countries on maternal, infant and child health outcomes?
- What are the impacts of nutrition counselling interventions on maternal, infant and child health outcomes across participants' PROGRESS+ characteristics?

## 3 METHODS

### 3.1 Criteria for considering studies for this review

#### 3.1.1 Types of studies

We will conform to the Cochrane Collaboration's Effective Practice and Organisation of Care (EPOC) criteria for the selection of studies (EPOC, 2017). We will include individual and cluster randomised controlled trials (RCTs), nonrandomised controlled trials (NRCTs), controlled before and after (CBA) studies as well as interrupted time series or repeated time measures studies (ITSs). We will follow EPOC guidance and only include ITS with three data points before and three data points after the intervention (EPOC, 2017). We will exclude cross sectional studies. Studies that do not include a control group will be excluded from the review as it is difficult to attribute causation with that study design.

#### 3.1.2 Types of participants

Participants include low risk pregnant women (15–49 years. with no active pregnancy‐related complications that require referral for additional management or specialist care) in different stages of gestation (in low‐ and middle‐income countries, as defined by the World Bank country income group categories at the time of the study's conduct.

#### 3.1.3 Types of interventions

The objective of this systematic review is to identify, appraise and synthesise the best available evidence on the effectiveness of nutritional counselling and education interventions in improving maternal and neonatal/infant health outcomes, and assess the differences in effect sizes across participants' PROGRESS+ characteristics.

The primary comparison is nutrition counselling versus no nutrition counselling. Studies that compare nutrition counselling to another intervention will be included and analyzed separately.

Support *could be* provided in addition to counselling as follows:

1. Providing nutritious food to malnourished individuals, based on anthropometric entry and exit criteria (low body mass index or low mid‐upper arm circumference).
2. Providing additional nutrient supplementation (e.g., micronutrient supplements).
3. Educate individuals on a variety of activities that aim to give them tools to meet their basic needs, including food, so that they do not have to rely on long‐term income transfers or food assistance.

The support must be present in both study arms for the study to be included as we aim to assess the efficacy of nutrition counselling.

#### 3.1.4 Types of outcome measures

We plan to assess the following outcomes.

##### Primary outcomes

Maternal Health:

- Mortality (up to 6 weeks postpartum).
- Anaemia (haemoglobin lower than 110 g/L) during and post intervention.
- Iron deficiency (as defined by study authors).

**Maternal behaviours**:

- Intent to breastfeed (as defined by study authors).
- Timely initiation of breastfeeding (proportion of women who initiated breastfeeding within one hour of birth).
- Adherence to Iron supplement consumption (proportion of women who reportedly consumed iron supplements during pregnancy).
- Adherence to nutrition counselling (as reported by study authors) (WHO, 2016).

**Neonatal health**:

- Stillbirths (death after 20 week's gestation and before birth).
- Preterm birth (before 37 week's gestation).
- Perinatal mortality (as defined by study authors).

Secondary outcomes

**Maternal Health**:

- Gestational weight gain (kg).
- Haemoglobin concentration post intervention.
- Haemorrhage (as defined by study authors).
- Mode of delivery (c‐section vs. vaginal).

**Maternal behaviours**:

- Dietary intake during pregnancy (kcal/day).
- Dietary diversity during pregnancy (macronutrients).

**Neonatal health**:

- Low birthweight (<2500 g).
- Small for gestational age (as defined by study authors).

We will include studies irrespective of whether they report outcomes of interest. We also recognise that some outcomes may be defined differently by different studies. For example, perinatal mortality is defined by mortality within first 4 weeks after birth but some studies may use a different definition. Similarly, adherence could be defined by participation in all counselling sessions or participation in a minimum number of sessions. We will collect details on the definition of all outcomes to decide whether the construct being measured is sufficiently similar, based on clinical expertise, to be included in meta‐analysis.

###### 3.1.4.1 Impact of intervention on health equity

To assess the impact of nutrition counselling on health inequalities, we will examine the effects of nutrition counselling across socially stratifying factors, if they are reported by authors. We will use the acronym PROGRESS+ to identify characteristics which may lead the population to being socially disadvantaged. PROGRESS is short for: Place of residence, Race/ethnicity/language, Occupation, Gender/Sex, Religion, Education, Socioeconomic status, and social capital/resources, personal characteristics (i.e., age, disabilities), relationship features (i.e., exclusion from school, parent drug use) and time‐dependent relationships (i.e., leaving the hospital or other times when an individual might be temporarily disadvantaged (O'Neill et al., 2014). We will report the findings of all these subgroup analyses as conducted within the studies. If there is data on subgroup analyses across the same PROGRESS‐Plus factor for more than one study, we will combine these using meta‐analysis to assess across study effects.

###### 3.1.4.2 Other eligibility criteria

Language and date of publication will not be restrictive criteria for our review. We will further translate studies identified in non‐English languages. We will also include protocols, peer‐review conference abstracts and studies in grey literature in our review and they will be classified as awaiting classification.

Adaptations to the protocol will be discussed with team members, documented and reported as a discrepancy from the protocol in the systematic review.

### 3.2 Search methods for identification of studies

We aim to search the following electronic bibliographic databases for relevant records: Medline via Ovid, Embase via Ovid, PsychInfo via Ovid, CINAHL via EBSCO, and the Cochrane CENTRAL Register of Controlled Trials via Ovid from date of database conception (Medline 1946, EMBASE 1974, PsychInfo 1967, CENTRAL 1996, and CINAHL 1961) to June 22, 2021. Furthermore, we will hand‐search reference lists of included studies and all relevant reviews identified by our search to ensure literature search saturation. We will seek consultations from content experts in the fields of nutritional counselling and health literacy in low‐ or middle‐income countries for any missing records. Moreover, we will search PROSPERO for any registered systematic reviews that have been recently published and hand‐search their reference lists for relevant records. Finally, we will search electronic registries of clinical trials such as clinicaltrials.gov and the WHO International Clinical Trials Registry for any recently published trials not captured by our search.

#### 3.2.1 Electronic searches

A comprehensive search strategy will be developed in consultation with a health science librarian with expertise in systematic review searches and will be adapted to the syntax and subject headings of each of the electronic databases that we plan to search. A combination of indexed terms, database‐specific and MeSH headings, as well as free text keywords will be used. Please see Appendix 1 for our search strategy. Keywords used to develop our search strategy include variations of the following: “Nutrition”, “Counselling”, “Education”, “Program”, “Communication”, “Diet”, “prenatal/perinatal care”, “Nutrition therapy” and “Pregnant women”.

We will filter out any editorials, comments, or personal communications to ensure that we only capture peer‐reviewed trials on our topic on interest.

#### 3.2.2 Searching other resources

We will further scan references of included studies in systematic reviews and unpublished studies or reports that satisfy our eligibility criteria using a focused google search, as well as the following grey literature sources:

- International Food Policy Research Institute.
- Alive and Thrive website.
- World Health Organization e‐Library of Evidence for Nutrition Actions (e‐LENA).
- Sight and Life Library.

### 3.3 Data collection and analysis

#### 3.3.1 Description of methods used in primary research

A broad range of intervention designs can be expected to be used as part of the studies identified in this review. They may include, home or clinic visits, group or individual counselling sessions and variety of intervention content.

#### 3.3.2 Selection of studies

Two review authors (A. B., A. S., O. D. or J. J.) will independently screen records yielded by our search against our inclusion/exclusion criteria using their titles and abstracts. To do so, we will use Covidence reference manager software (Covidence). Subsequently, eligible records will be screened by full text, in duplicate and independently, to evaluate if they truly meet our inclusion criteria. Discrepancies between reviewers will be resolved by consensus or with the help of a third member of the research team (V. W.) when required. We will prepare a PRISMA study selection chart (Moher et al., 2009) along with references for excluded studies for transparent reporting.

#### 3.3.3 Data extraction and management

Data extraction will be conducted independently and in duplicate. Conflicts will be resolved by consensus or with the help of a third member of the research team (V. W.) when required. Also, if the authors of primary studies need to be contacted about missing information, they will be reached out to by their primary contact information for a maximum of three attempts without reply in between. A standardised data extraction framework will be developed in consultation with content and health equity experts using excel sheets. See Supporting Information Appendix S2 for a preliminary data extraction sheet. To ensure the validity of our data extraction framework and increase its compatibility with our analysis objectives, we will pilot test the extraction process with a random sample of *n* = 5 included records and revise the process accordingly.

Reviewers will extract the following variables:

(1) Study identifiers: such as name of authors, date of publication, journal, volume, and page number if needed.
(2) Study methodology: objectives, study design, methodological details such as processes for randomisation, allocation and blinding, target population, recruitment and sampling procedures, setting, participant eligibility criteria, participant baseline characteristics, sample size per arm at baseline.
(3) Intervention description: name, nature, components (e.g., timing, frequency, route of delivery, empowerment approach elements), and details of the comparison intervention.
(4) Outcomes: Definitions, instrument and scale interpretation, timing of outcome measures, and adverse events.
(5) Results: Participant attrition rate, categorical data, continuous data, between‐group estimates.
(6) Author conclusions, funding and conflict of interest.

The data extraction form will emphasise the separate extraction of health and behavioural outcomes to ensure consistency with data reporting.

“Throughout data extraction we will assess whether outcome data is stratified by PROGRESS+: place of residence, race and culture, occupation, gender and sex, religion, education, socioeconomic status, social capital, personal characteristics (i.e., age, disabilities), relationship features (i.e., exclusion from school, parent drug use), and time‐dependent relationships (i.e., leaving the hospital or other times when an individual might be temporarily disadvantaged) (O'Neill et al., 2014).

#### 3.3.4 Assessment of risk of bias in included studies

Risk of bias of individual studies will be assessed by two independent reviewers at the study level using R.O.B 2.0 tool. At the study level, the Cochrane collaboration tool for assessing risk of bias will be used when assessing bias for RCTs (Higgins, Jelena, et al., 2019). The domains include sequence generation, allocation concealment, selective reporting, blinding of participants/personnel/outcome, selective outcome reporting, and incomplete outcome data. All biases will be assessed by providing a judgement (high, low, some concerns) on individual elements from the five domains. In the likelihood of assessing risk of bias in nonrandomised studies of interventions, the Newcastle‐Ottawa Scale tool will be used to assess the risk of bias in nonrandomised studies (Wells et al., 2000). This tool will be used to assess any biases in the selection of participants, comparability of cohorts, and adequacy of outcome assessment. All Judgements of biases will be made independently by two reviewers. Disagreement will be resolved through discussion or consulting with the study supervisor (V. W.). To provide a graphic representation of bias between and within studies, we will use RevMan 5.3 software (RevMan, 2014). Modified EPOC risk of bias will be used to critically appraise interrupted time series and CBA studies (EPOC, 2017). This tool assesses protection against contaminations, recruitment bias, and detection of analysis errors in studies by cluster allocation instead of individuals. To minimise risk of bias in individual studies, experimental studies will be given priority over observational studies to prevent subjective analysis and interpretation.

#### 3.3.5 Measures of treatment effect

Within each of our outcomes of interest, we will assess heterogeneity across the type of intervention to decide if it is sensible to pool the data together. We will conduct all possible meta‐analyses of effect estimates for RCTs and NRCTs separately while accounting for differences in outcomes, interventions and comparators. ITS studies will be analyzed using the methods recommended by EPOC (EPOC, 2017). Continuous outcomes, such as iron deficiency, anaemia/haemoglobin concentration, or birthweight, will be analysed using mean differences in change from baseline as possible. In the availability of baseline and end‐point data, we will calculate the change from baseline and associated standard difference as provided in the Cochrane handbook for systematic reviews of Interventions (Higgins, Li, et al., 2019). All continuous outcomes will be accompanied by estimates of statistical significance such as SDs, SEs of the mean, and 95% confidence intervals (CIs). Dichotomous outcomes, such as the rate stillbirths, perinatal and neonatal mortality, will be analysed using relative risk measurements such as risk ratios. Similarly, all dichotomous effect estimates will be accompanied by statistical significance estimates such as 95% CI and *p* values. When more than one publication report effect estimates from the same population receiving the same intervention, we will report effect estimates that encompasses a larger sample size.

#### 3.3.6 Unit of analysis issues

For cluster randomised trials, we will assess unit of analysis errors (e.g., no adjustment for clusters made). If errors are detected, we will inflate the SD using an intra‐cluster correlation coefficient in accordance with the Cochrane handbook for systematic reviews of Interventions (Deeks et al., 2019). In the presence of dichotomous outcomes, we will adjust the numerator and denominator for unit of analysis errors.

For studies with multiple arms, we will select all arms that fill the inclusion criteria and still provide a “control” arm. In turn, we will analyze each arm in comparison to the control arm separately. If more than one arm provides two‐way interactive communication with the participants and there is no control remaining, we will assign the intervention arm as the arm that is considered to have the most interactive two‐way communication with the participants. The Control arm will be the arm considered to have the least interactive two‐way communication with the participants. The unit of analysis will be per individual randomised in the study.

##### Criteria for determination of independent findings

Articles reporting the same study in multiple publications will be linked and coded as one study to avoid the double counting of study participants. All the analyses will be conducted according to the outcome as previously described. If an outcome is reported in different metrics, we will perform unit conversions to permit pooling of data.

#### 3.3.7 Dealing with missing data

Whenever data from included primary studies are not reported or lack details (e.g., missing details on variation such as SD, number of participants) or missing details on PROGRESS+ factors, we will contact authors to obtain such missing data. Three attempts will be made to contact the corresponding authors for missing data with three working days between the attempts. If authors do not respond or are not able to provide missing data, we will report effect estimates as reported only. Values and SDs for missing data will not be imputed. Unavailable SDs will be calculated using other methods such as CIs and exact *p* values using the formulae provided in the Cochrane handbook for systematic reviews of Interventions (Page Matthew et al., 2019). Studies with no quantitative results will not be included in our analysis.

For continuous outcomes, we will use intention to treat analysis method, using the number of individuals randomised into the study, including missing individuals. For dichotomous outcomes, we will also use intention to treat when analysing our data, thus the total number of participants in the study will be used as the denominator, assuming that the event did not occur in the missing individuals (Higgins, Li, et al., 2019). Sensitivity analysis will be conducted when primary studies report per‐protocol analyses.

#### 3.3.8 Assessment of heterogeneity

Statistical heterogeneity will be assessed using the *I* ^2^ statistic and *χ* ^2^ test of independence.

#### 3.3.9 Assessment of reporting biases

Funnel plots will be used to assess the risk of publication bias in our analyses with 10 or more studies to prevent biasing the estimated between‐study heterogeneity variance (Higgins, Thomas, et al., 2019). We will use RevMan 5.3 software to visually create the funnel plots (RevMan, 2014).

#### 3.3.10 Data synthesis

We anticipate a certain degree of unreported heterogeneity in the delivery of the intervention in low‐ or middle‐income countries, therefore we will use random effect models in our analysis. All pooled results will be reported using forest plots and pooled effect estimates.

We will not pool data from randomised and nonrandomised study designs.

#### 3.3.11 Subgroup analysis and investigation of heterogeneity

If applicable, we will conduct subgroup analyses for maternal health, neonatal/infant health and maternal behavioural outcomes across the following:

- Frequency or type of intervention (web based, one‐on‐one or group).
- Specific equity characteristics across the PROGRESS+ criteria (Education, socioeconomic status and age are considered the most important for this question).
- Time of commencement of the intervention by gestational age.

Subgroup interaction will be tested for in Review Manager 5.3. We will document if any of these subgroups were conducted within the studies and report them in our review.

*I* ^2^ value with a cut‐off of 0.75 or higher will be considered for subgroup based on any clinically important differences in study populations, characteristic of interventions, nature of comparator groups, and outcome measurements (McKenzie Joanne & Brennan Sue, 2019).

##### Empowerment analysis

We will classify included studies by the extent to which they apply a complete empowerment model. Included studies will be classified according to the following categories: (1) Complete empowerment model (i.e., including agency and opportunity structure activities), (2) Partial empowerment model (i.e., including agency‐related activities only), or (3) Unclear empowerment approach. Classification decisions will be based on the primary author's description of the intervention.

Given a sufficient number of included studies, we will conduct the following subgroup analyses: (1) complete empowerment model versus unclear empowerment approach, (2) complete empowerment model versus partial empowerment approach, (3) partial empowerment model versus unclear empowerment approach. If there are too few studies for subgroup analysis, we will provide a narrative synthesis comparing the effects across the three categories.

#### 3.3.12 Sensitivity analysis

Sensitivity analysis will be conducted across risk of bias (generation of sequence, allocation bias and protection against contamination) and for methodological imputations (e.g., adjustment for unit of analysis errors).

##### Treatment of qualitative research

We do not plan to include qualitative research.

#### 3.3.13 Summary of findings and assessment of the certainty of the evidence

We will tabulate outcome measures in GRADE summary of findings (SoF) tables aggregated in the following categories: Maternal health outcomes, Neonatal/Infant health outcomes, Maternal behavioural outcomes with seven outcomes per SoF table. The table will be generated as per recommendations in the Cochrane Handbook and will include:

(1) Primary and secondary outcomes of the review.

(2) Measures of absolute magnitude of intervention effect.

(3) Number of participants.

(4) Grade of the overall quality of the body of evidence.

(5) Comments that aid the interpretation of the results.

Certainty of evidence will be evaluated using GRADE methodology (Atkins et al., 2004) to assess our certainty in the evidence. We will present our certainty levels as either high, moderate, low or very low. The results of each outcome measure will be assessed against eight criteria. The following five criteria are considered for possible downgrading the quality of evidence: study quality (risk of bias), consistency (consistency between included studies), precision of results, directness (same population, intervention and outcomes as we desire) and reporting bias. Three criteria may upgrade the level of certainty: strength of associations between intervention and outcome; size of the dose‐response effects; and where all plausible confounders would have reduced the effect. We expect that there might be challenges with pooling of results. If this occurs, we plan report the rating of the certainty of evidence using a narrative summary using the GRADE approach (Hassan Murad et al., 2017).

## CONTRIBUTIONS OF AUTHORS

- *Content*: Jessica C. John, Sarah Wuehler, Seth S Klobodu, Manoj Kumar Raut, Jennifer Busch‐Hallen, Erika Ota, Sarah Rowe, Chowdhury Jalal, and Jacqueline K. Kung'u.
- *Systematic review methods*: Vivian Welch, Omar Dewidar, and Ammar Saad.
- *Statistical analysis*: Omar Dewidar, Vivian Welch, and Alison Riddle.
- *Information retrieval*: Omar Dewidar, Ammar Saad, Aqeel Baqar, and Jessica C. John.
- *Content expert*: Jennifer Hatchard.

## DECLARATIONS OF INTEREST

The authors declare to have no competing interests. Jennifer Busch‐Hallen, Chowdhury S. B. Jalal, Jacqueline K. Kung'u, Manoj K. Raut, Sarah Rowe, Sara Wuehler are employees of Nutrition International (NI), which provided funding to the implementation of the study. However, funding was provided after the study was conceptualised and the proposal was drafted.

## PRELIMINARY TIMEFRAME

Submission of a draft review: September 1st, 2020.

Submission of final review: November 30th, 2020.

## PLANS FOR UPDATING THIS REVIEW

Once the review is completed, it will be updated every 5 years.

The Senior author will be responsible for updating the review.

## EXTERNAL SOURCES

- Nutrtion International, Canada.

This project is funded by Nutrition International.

## Supporting information

Supporting information.

## Footnotes

^1^
The presence of agency and opportunity structure‐related activities will not be used as inclusion criteria, but rather, will be used to conduct subgroup analyses comparing nutrition counselling interventions that do and do not apply a complete empowerment model.
