# Supplementary material for: PROTOCOL: Effectiveness of nutrition counselling for pregnant women in low‐ and middle‐income countries to improve maternal, infant and child behavioural, nutritional and health outcomes: A systematic review
Source: Campbell Syst Rev. 2021 Nov 9;17(4):e1202. doi: 10.1002/cl2.1202 (PMC8988835; doi:10.1002/cl2.1202)
Supplement: Supplementary file 1 — Supporting information. [file CL2-17-e1202-s001.docx]

# Feedback

# Appendices

## 1 Appendix A: Medline search strategy

1. pregnancy/ or pregnancy outcome/ or pregnancy, high-risk/ or pregnancy, unplanned/ or pregnancy, unwanted/ or prenatal nutritional physiological phenomena/

2. prenatal care/

3. perinatal care/

4. pregnant women/

5. pregnan*.ti,ab,kf.

6. ((perinatal or prenatal) adj2 care*).ti,ab,kf.

7. 1 or 2 or 3 or 4 or 5 or 6

8. Nutrition Therapy/

9. nutritionists/

10. counseling/ or health education/ or patient education as topic/ or prenatal education/ or intervention/ or communication/ or program/

11. nutritional physiology phenomena/ or diet/

12. 10 and 11

13. ((nutrition* or diet*) adj3 (counsel* or educat*)).ti,ab.

14. 8 or 9 or 12 or 13

15. 7 and 14

16. (Editorial or comments or Letter).pt.

17. 15 not 16

## 2 Appendix B: Data extraction items

1. Extractor ID (initials)
2. Citation of the Study
3. Any accompanying studies/ publications related to this trial?
4. Description of the study
   1. Objective and hypothesis
   2. Study design
   3. Target population
   4. Setting (clinic, county, neighbourhood, city, country)
5. Study methodology
   1. Recruitment and sampling procedures
   2. Participant eligibility criteria
   3. Randomization and allocation to study arms
   4. Blinding and practices addressing biases
   5. Data collection and analysis
6. Baseline assessment
   1. Baseline characteristics (Any characteristics reported by the PROGRESS+ criteria)
   2. Any difference in baseline characteristics between groups? Any actions to address that?
7. Description of the intervention and outcome measurements
   1. Name of the intervention
   2. Description of the intervention (Components, dose, timing, frequency, route of delivery, elements of empowerment)
   3. Comparison/ control group
   4. Description of the outcome measured (What outcome are they measuring? What instrument/ tool are they using? What scale is this instrument using? When/how often are they measuring this outcome?
   5. Describe any stratification of results by the PROGRESS+ criteria
8. Results
   1. Participant follow-up (How long the follow-up? Drop-outs? Are drop-outs balanced between groups?
   2. Categorical outcomes (report effect estimates, confidence intervals, and p values when possible)
   3. Continuous outcomes (report effect estimates, confidence intervals, and p values when possible)
   4. Equity-specific findings (report effect estimates, confidence intervals, and p values stratified by PROGRESS+ criteria when mentioned)
9. Miscellaneous
   1. Study limitations as reported by authors
   2. Study funding as reported by authors
